# Communication in disasters to support families with children with medical complexity and special healthcare needs: a rapid scoping review

**DOI:** 10.3389/fpubh.2024.1229738

**Published:** 2024-03-13

**Authors:** Neale Smith, Meghan Donaldson, Craig Mitton, Esther Lee

**Affiliations:** ^1^Centre for Clinical Epidemiology and Evaluation, Vancouver Coastal Health Research Institute, University of British Columbia, Vancouver, BC, Canada; ^2^Complex Care Program, British Columbia Children’s Hospital, Vancouver, BC, Canada; ^3^Canuck Place Children’s Hospice, Vancouver, BC, Canada; ^4^Department of Pediatrics, University of British Columbia, Vancouver, BC, Canada

**Keywords:** children with medical complexity, disaster, emergency response, communication, review

## Abstract

Disasters can disrupt normal healthcare processes, with serious effects on children who depend upon regular access to the health care system. Children with medical complexity (CMC) are especially at risk. These children have chronic medical conditions, and may depend on medical technology, like feeding tubes. Without clear, evidence-based processes to connect with healthcare teams, families may struggle to access the services and supports they need during disasters. There is limited research about this topic, which has been pushed forward in importance as a result of the COVID-19 pandemic. The authors therefore conducted a rapid scoping review on this topic, with the intention to inform policy processes. Both the peer-reviewed and gray literatures on disaster, CMC, and communication were searched in summer 2020 and spring 2021. Twenty six relevant articles were identified, from which four main themes were extracted: 1. Cooperative and collaborative planning. 2. Proactive outreach, engagement, and response. 3. Use of existing social networks to connect with families. 4. Return to usual routines. Based on this review, good practices appear to involve including families, professionals, other stakeholders, and children themselves in pre-disaster planning; service providers using proactive outreach at the outset of a crisis event; working with existing peer and neighborhood networks for support; employing multiple and two-way communication channels, including social media, to connect with families; re-establishing care processes as soon as possible, which may include virtual connections; addressing mental health issues as well as physical functioning; and prioritizing the resumption of daily routines. Above all, a well-established and ongoing relationship among children, their caregivers, and healthcare teams could reduce disruptions when disaster strikes.

## 1 Introduction

During times of disaster or crisis, normal patterns of care can be disrupted, perhaps for quite a period of time, with potentially serious or deadly effect on children who depend upon regularly scheduled and uninterrupted access to the health care system. Particularly at risk are children with medical complexity (CMC), who have chronic medical conditions often with technology dependence (e.g., feeding tubes). These children represent about 1% of the pediatric population but require approximately 30% of pediatric health care resources, including hospital and community care (1). For instance, in Canada, among this population 68% are reported to require at least one emergency department visit per year, and 36% are hospitalized at least once per year. The average number of hospitalizations for a CMC annually is 2.5, with an average hospital stay of 21 days (2).

The families of these children rely on teams of health care professionals, spanning the hospital and the community, to partner in their care. However, lack of clear, standardized and evidence-based processes for communication among families and healthcare teams during disaster-related disruptions can make it very challenging for families to maintain needed access to services and supports.

Given that relatively little is known about this topic, and that the issue has been pushed to the forefront due to the COVID-19 pandemic, the authors undertook to synthesize available evidence as a beginning guide for policy discussions. We employed a rapid scoping review approach to knowledge synthesis. Rapid reviews provide “actionable and relevant evidence in a timely and cost-effective manner” (3), p. 3 and “scoping studies… map rapidly the key concepts underpinning a research area and the main sources and types of evidence available” (4), p. 194. Knowledge in a broad range of forms is expected to be relevant.

### 1.1 Key concepts

#### 1.1.1 Children with medical complexity

One of the challenges in this review was determining if different studies included comparable populations, and/or if the communication challenges were similar or different across settings and among specific groups of professionals or pediatric patients. The broadest term for the population of interest encountered with the literature was perhaps CAFN, or Children with Access and Functional Needs (5), which “is now preferred to the term ‘special needs,’” (6), p. 70 as being more inclusive. Boon et al. note that children with disabilities, and children with special health care needs, are not necessarily synonymous terms (7), p. 232; presumably not all children with disabilities require substantial additional on-going medical care. It is more common to consider persons with disabilities as a sub-group within this larger population.^1^ Kailes and Lallor present the CMIST framework, which breaks functional need into five sub-categories: communication (C); maintaining health (M); independence (I); support, safety, and self-determination (S); and transportation (T) (8).

Terms more specific to the health sector and in relatively common use include 1. CSHCN - Children with Special Health Care Needs - which is typical nomenclature in the United States and 2. CMC - children with medical complexity.

CSHCN is formally defined as “those who have or are at increased risk for a chronic physical, developmental, behavioral, or emotional condition and who also require health and related services of a type or amount beyond that required by children generally,” as cited in (9), and would include chronic conditions such as diabetes or asthma. US estimates are that this includes 15% of all children (9). CMC can be seen as a subset of CSHCN (10, 11). The term is defined by Cohen et al. as “children who are the most medically fragile and have the most intensive health care needs.… and includ[ing] children who have a congenital or acquired multisystem disease, a severe neurologic condition with marked functional impairment, or patients with cancer/cancer survivors with ongoing disability in multiple areas” (10). According to Cohen et al., “CMC are … children with characteristic patterns of needs, chronic conditions, functional limitations, and health care use” (10). In their systematic review, Hipper et al. used the definition, “children with chronic, severe health conditions and major functional limitations” (12), p. 179.

More expansive definitions of special needs children, such as the inclusion of those with intellectual or behavioral challenges, make the population more difficult to identify in advance (13). On the other hand, there are also studies which use more restrictive definitions limiting their scope to subsets of CMC, and so implications for supports and communication needs during disasters might not be generalizable to the larger group of CMC. Examples include Hoffman et al. who use both CMC and the term VPP (vulnerable pediatric patient), defined as being those who are technology-dependent (14). In a 2009 paper, Uscher-Pines et al. focus upon the needs of children who require specialized forms of transportation (e.g., who use wheelchairs) (15). Rogozinski et al. employ the term PCCI, for children with pediatric chronic critical illness, or in other words that sub-group requiring the most clinical intervention, supports and resource use (16).

#### 1.1.2 Disasters

For the purposes of this paper, our working definition of disaster is that of the International Federation of Red Cross/Red Crescent Societies: “A sudden, calamitous event that seriously disrupts the functioning of a community or society and causes human, material, and economic or environmental losses that exceed the community’s or society’s ability to cope using its own resources”.^2^ Five factors feature in formal typologies of disaster events: (a) type of disaster (natural or human-caused), (b) duration, (c) degree of personal impact, (d) potential for occurrence, and (e) control over future impact (17). Thus, disasters will vary by scale (wide-spread or localized), and duration (that is, they can occur in a short time span and be quickly resolved, or they may last over a prolonged period of time); they can come on suddenly, or evolve slowly over time, such as with the COVID-19 pandemic. They can be forewarned and anticipated, or occur relatively unexpectedly or with little lead time to prepare. Most parts of the world are subject to some form of recurring disaster threat, with the specific type (e.g., earthquake, wildfire etc.) varying by geography and geopolitical circumstances.

Highly destructive events will affect the health system’s ability to provide usual or alternative resources on a timely basis, and families may be displaced from their homes and communities for brief or extended periods of time. In addition to any threats to physical health which this might pose, displaced persons will experience a range of psycho-social ill effects and may need to rebuild their networks of social support (18–20). Key to disaster as we understand it, then, is that it is a mass event (not an individual medical crisis) and one which in addition disrupts the ability of individuals and families to access and receive care for a period of hours, days or longer.

#### 1.1.3 Communication

Communication similarly can vary in a number of ways. For example, it can be between professionals and a family or caregiver of a child with medical complexity (CMC) or peer-to-peer between professionals or among families. It may be one-way or two-way; direct or mediated (e.g., through an administrative assistant to parents, or through a caregiver to the children themselves); and need to involve only two parties, or multiple persons and organizations. It might be a one-time event, or involve regular and on-going contact and follow-up. Information can be transmitted orally, or in a written or recorded format; and delivered in real-time or exist as static resources that can be accessed asynchronously. It can be reactive, or proactively involve pushing information or reaching out and contacting patients during or following an emergency. It can communicate accurate information, or address and correct mis-information. It can be individualized and tailored to an individual patient, or employ standard messaging in mass or social media forms. This description is intuitive rather than based on a particular model of human communications; thus, this list may not be exhaustive.

There is also variability in individuals’ ability to receive materials by certain channels: this includes physical restrictions, e.g., hearing/vision impairment, but also social-technological barriers (e.g., lack of internet access or cell phone coverage or inability to communicate in the main language of community). Such factors will need to be accounted for when determining what will be effective means and methods of communication during disasters.

We might also presume that the nature of communication challenges and needs would vary across types of disaster situation. One difference is the number of CMCs who would be impacted at once (placing different levels of demand upon professionals’ time and attention). And of course, professionals themselves may be directly affected or displaced to different degrees. CMCs also have different types of needs (e.g., mechanical ventilation, specialized transportation, or specific nutrition) which may be provided at home, or require visits to a medical clinic or other facility. This can affect the content of what communication is needed during a disaster.

These ideas are summarized in generalized principles for effective disaster-related communication, as stated by Kailes and Lollar:

“Information [should] be real, specific, and current… relevant information should be developed in partnership with people who live with disabilities… [and] be made available in accessible, [multiple] and usable formats.” (8), pp. 258–259.

The characteristics of each of the three main concepts, as given here, were drawn upon to map the aspects of communication about which each relevant study identified in the review might provide useful data or lessons, as discussed in methods and results. Ultimately, this aims to serve the purpose of this project, to better inform clinicians and policy makers about the unique needs of CMC which must be addressed during crises, so that they can improve both preparation and response.

## 2 Methods

Standard approaches to conducting a rapid scoping review involve multiple steps, (21–23). We carried out this review following the six steps defined below. I. Define and align the objective(s) and question(s). II. Develop and align the inclusion criteria with the objective(s) and question(s). III. Search for the evidence. IV. Select the evidence. V. Extract the evidence. VI. Analyze the evidence.

**Figure 1:**
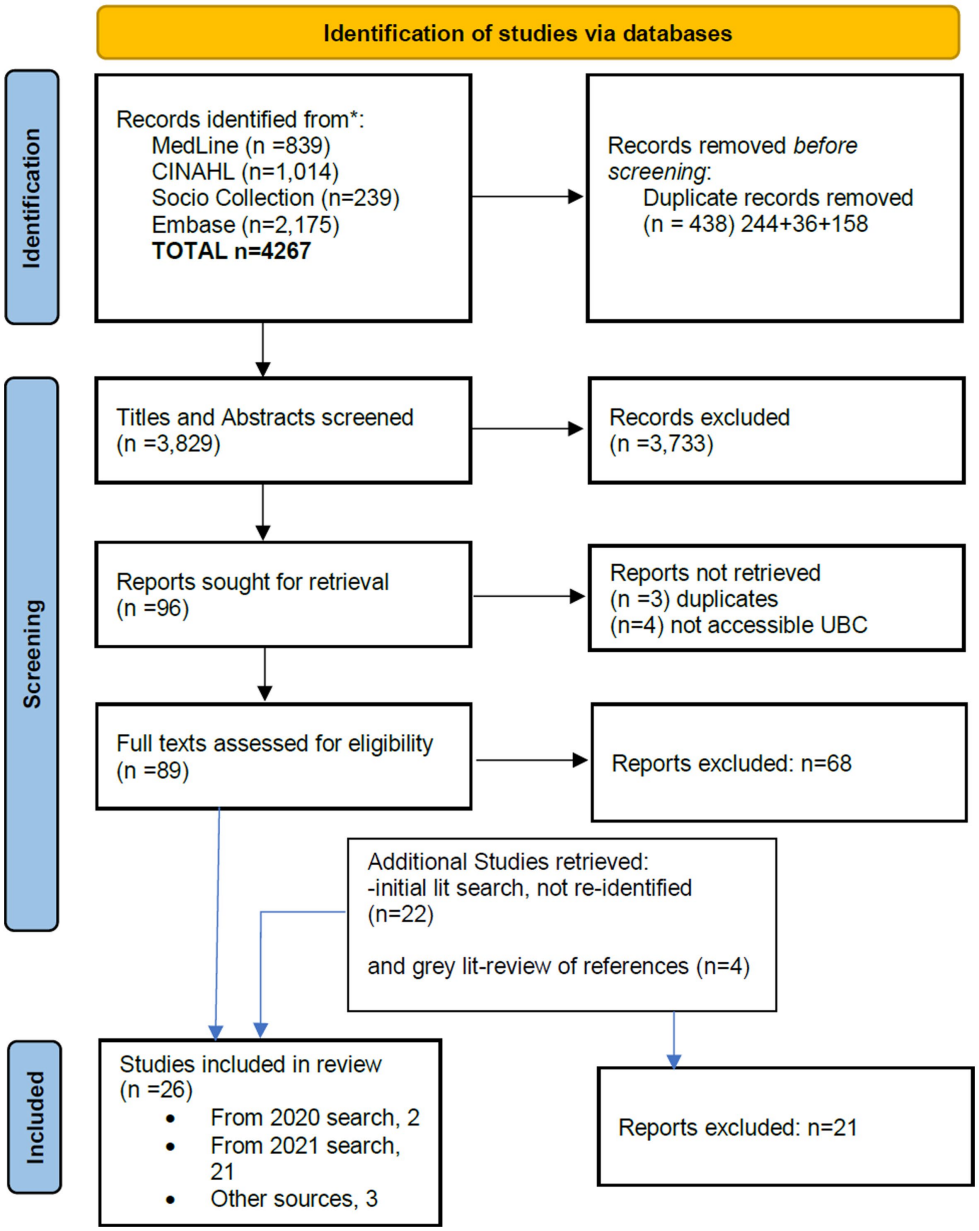
PRISMA diagram. Adapted with permission from Page et al. (24), licensed under CC BY 4.0, http://prisma-statement.org/prismastatement/flowdiagram.aspx.

**Figure 2:**
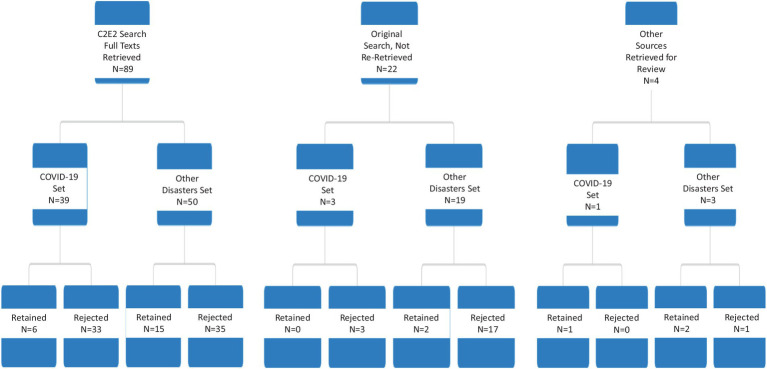
Disposition of full-texts.

- Research question: Our research question was, ‘What are the best ways in which the health system can communicate during times of crisis or disaster with families of CMCs?’ This research area was broadly addressed by a previous scoping review on disaster information needs for CMC published in 2018 (12). Most of the publications identified in this review centered on wide scope of disaster planning and emergency preparedness, rather than focusing on communication during crises and in the recovery and rebuilding phases. Our review particularly investigates if further information has become available in the latter two areas. As well, we expect that use of social media, and the COVID-19 pandemic will have generated additional publications not thoroughly considered before. Our present review is therefore an extension, rather than updating alone, of previous work.
- Inclusion and exclusion criteria: these are expressed below in the form of the PICOS elements --population, intervention, comparator, outcomes, and study types.
  - Population: relevant populations include any or all of three groups – (a) children with medical complexity (CMC) and/or their families and caregivers; (b) health and education professionals proving services to these children; (c) emergency responders who may encounter these children during disaster situations. Included papers were required to address both disaster/public emergency/mass casualty situations and children with special healthcare needs/medical complexity. See the section on search strategy below, and the detailed Appendix A, for the operationalization of these concepts. Children *per se* were not defined as a vulnerable population for this paper; the focus of the review is upon children with special needs who are at baseline community-dwelling, and so papers focused upon neo- or perinatal institutional care were excluded. We did not limit inclusion to only CMC, but included those with other functional needs or disability, so long as the findings appeared to be broadly applicable for the CMC population. Papers focused primarily upon planning for or responding to individual medical emergencies were excluded, as were papers which only described the physical or mental health effects of disaster.
  - Intervention: our focus is on communication strategies employed among members of these three groups. This includes, but is not limited to, studies which describe lines of communications between healthcare providers and the families of CMC, methods of maintaining access to needed services, communication protocols and messaging and their efficacy during disasters, and reports from professionals (including doctors, nurses, social workers, educators and school support personnel) on their experiences in coordinating disaster communications.
  - Comparator: Given the diversity of approaches eligible for inclusion, and the unlikelihood that there will be total absence of communication with CMCs during an emergency, no comparator was specified for the review.
  - Outcomes: any assessment of the effectiveness of communication among these three groups, in terms of minimizing impacts upon physical, emotional and social well-being during disasters, and ensuring uninterrupted access to necessary medical care.
  - Study type: As the review is interested in including publications written by or with direct involvement of family members or caregivers, this necessitates inclusion of paper types and sources normally excluded from systematic reviews, such as Hipper et al. (12). Research protocols and individual patient case reports were excluded; but otherwise most article types were eligible for inclusion. Only English language papers were included.
- Search strategy: Two searches were run for the project. A health information specialist at BC Children’s Hospital ran a search of Medline, CINAHL and gray literature in summer 2020. 59 publications were retained from this search for possible full text review. Based upon examination of these papers, a revised search was developed and completed by the Center for Clinical Epidemiology and Evaluation (C2E2)‘s health information specialist in spring 2021 in Medline, CINAHL, Embase, and Sociology Collection. Search strategies are reported in Appendix A.
- Evidence selection: Titles and abstracts from the spring 2021 search were initially reviewed by one reviewer at C2E2. Those for which a clear inclusion or exclusion determination could not be quickly made were reviewed by a second reviewer, who used the same criteria to make a final determination as to whether or not full-text review seemed warranted. Articles identified for full text review were retrieved, where possible. Full texts were divided into two groups: COVID-19 related and other disasters. Articles in each group were read, and some further excluded at this point for not meeting inclusion criteria or being otherwise not relevant. After completion of this process, 26 articles were retained for data extraction. See Figure 1 for PRISMA diagram and Figure 2 for the disposition of full-texts within each category.
- Data extraction. Categories in the data extraction template included year of publication; country; study design/article type; whether or not CMC were the primary focus; intervention (if any); types of qualitative and quantitative data collected and reported (if any); type of disaster; stage of disaster; key results; and any general comments and judgments related to relevance for the research question. COVID and non-COVID papers were extracted in separate batches by different reviewers.
- Data analysis. Since very few of the articles were explicit about the role of communications in disaster response –i.e., there was little manifest content (25) -- we conducted latent content analysis, to identify and code blocks of text in which approaches to communication are alluded to, or can be seen occurring even if not remarked upon by study authors (26, 27). In particular we apply latent projective analysis (28), looking beyond the text itself and drawing upon our own understanding of health and communication theories. After draft analysis and reporting was completed, two patient partners, both parents of CMC, were engaged to provide feedback on the draft report summary and the embedded Vignette (see later); both were compensated for their time in accord with the funders’ guidelines (29). These parents provided feedback during a real-time virtual meeting and subsequently via email, and improvements to the write-up were made in consequence.

## 3 Results

### 3.1 Summary of main findings: descriptive results

**Figure 3:**
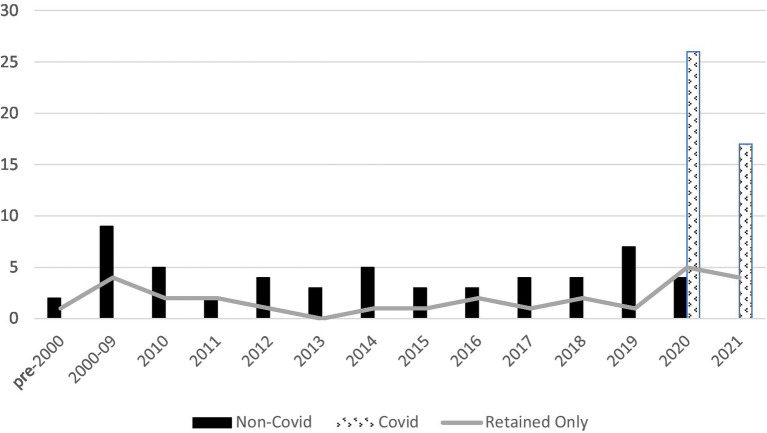
Full text articles retrieved, and retained, by year of publication.

A total of 26 full-texts were included in the review: 7 papers on COVID-19, and 19 papers on other forms of disaster or crisis. The following sections describe the findings from these 2 sets of papers; a narrative summary of each source is included in Appendix B. Countries represented were United States (*n* = 13), or 50%, followed by Japan (*n* = 3), New Zealand (*n* = 2), France (*n* = 2), Italy (*n* = 2) and one each from Greece, Turkey, the United Kingdom and Australia; this includes both empirical and non-empirical studies. (The earlier Hipper systematic review reported 81% of papers, or 22 of 27, to be from the United States context.) Considering publications by year (Figure 3) suggests a small but steady flow of articles potentially relevant to the topic of this review. Of the 26 retained paper, one-half (50%, *n* = 13) were published between 2017 and 2021; 5 were published between 2012 and 2016, and the balance (*n* = 8) were published more than 10 years ago. About one-quarter of papers (6/26) are published in journals or as a book specific to the field of disaster and emergency medicine, while the others target a range of generalist and specialist audiences of health professionals.

Twenty-one of the 26 papers were entirely or primarily about children with special needs. These were not limited to CMCs; for instance, some addressed children with sensory disorders, such as deafness (30, 31), developmental disabilities, including autism (32), and chronic diseases, e.g., diabetes (33). While it has been suggested that there may be structural program differences between care for children with a single defined illness or disease, and care for CMC, with the former focusing on disease management and the latter on care coordination (34), we deemed that any information about communication strategies in the context of disaster would likely be transferrable. The five remaining papers included targeted comments about this group within the context of a larger discussion, project or study.

Table 1 summarizes publications by disaster type and by the stage –planning, response or recovery – which is most substantially addressed within each.

**Table 1:** Retained papers by disaster type and stage.

	All hazards	Earthquake	Hurricane	Pandemic
Planning	8	4	0	0
Response	0	1	1	7
Recovery	1	3	1	0

The largest proportion of the reviewed papers (12/26 papers, or 46%) focuses upon disaster planning and preparedness, though the relative proportion is skewed by the COVID-related literature; in this, our review finds the same as Hipper et al. (12) (in that work, slightly less than half of retained studies, 14/27, focused exclusively on preparedness, and only 4 papers had no focus on preparedness). Table 1 also indicates, again consistent with Hipper et al., that much of the disaster planning and preparation literature is all-hazard. In this review, that category accounts for 8/26 (or 31%), compared with findings in Hipper et al. of 19/27 papers, or 70%.

Baker, Baker and Flagg note that the ‘all-hazards’ approach is recommended for disaster preparedness (35), p. 418 and that specific tailoring may be unnecessary, though by contrast, Chang et al. suggest that tailoring should be considered after initial disaster planning based on the all-hazards model (36). Drexel University’s Center for Public Health Readiness and Communication provides tailored checklists, because they heard this request from parents.^3^ Similarly, resources for talking with children after particular types of disasters (e.g., earthquakes, hurricanes and tornados) are offered by the Centre for Safe & Resilient Schools and Workplaces^4^ though these are not specific to CMC.

In the context of the authors’ location, British Columbia, Canada, earthquakes and tsunami, other floods and wildfires, avalanche or landslide may be the most likely natural disaster scenarios, along with pandemic disease outbreaks such as COVID-19.^5^

A variety of research designs are used in the retained publications; it is possible for a paper to use more than one of the listed designs, so the total exceeds 100%. This review found 19/26 papers (74%) to include original qualitative or quantitative research; Hipper et al.’s review (12) included 12/27 original research papers (44%).

- Survey = 13
- Case study/description = 7
- Commentary = 4
- Interventional = 3
- Literature review/synthesis = 3
- Qualitative design = 3
- Document review = 1

Where original data was collected, in most cases it was from the parents or caregivers of children with access and functional needs. In three cases, researchers worked directly with the children or youth. In some articles, the study population was not clearly described. In one case, websites and resource materials were the subject of data collection and analysis. Articles were directed at a variety of provider/practitioner audiences, including primary care physicians/medical homes, specialty care (e.g., nephrology, oncology), occupational therapists, speech language pathologists, social workers, school nurses and other educators, and emergency responders and transporters. The lead author in the majority of cases (*n* = 14) was an academic-clinician, i.e., someone working at a university or teaching hospital. For remaining papers, the lead authors were, respectively, academics working in a non-clinical university department (*n* = 5), community-based clinicians (*n* = 3), government employees (*n* = 2), not-for-profit organizations (*n* = 1) and parents (*n* = 1).

Communication-related content of the papers, whether manifest or (more commonly) latent, is categorized in Table 2. As the table suggests, there is some recognition of the value of proactive outreach at the time of a disaster, though the issue mostly is not evidently addressed. Most papers consider communication between health care professionals and families/caregivers, with a smaller number focused upon communicating with CMC directly. Typically, only one-way communication is described, though implicitly there is often back-and-forth among health professionals and families. Communication is typically in the form of mass or standardized products, with only a few papers describing approaches with some degree of targeting or tailoring to the specific circumstances of the families involved. Finally, while social media is a growing aspect of disaster response, only a few of the more recently published articles contain either brief or detailed description of how this can be or is used for communication during emergency or crisis circumstances.

**Table 2:** Aspects of communication reported in the retrieved papers.

Aspect	Assessment of how aspect treated within reviewed papers			
Proactive outreach at the time of disaster	Demonstrated 2	Called for 5	Not present 19
Directionality of communication	Between health professionals and families or caregivers 18	Explicitly and primarily directed at CMC 3	Peer-peer among families or communities 5
Messaging	Standardized messaging 7	Tailored or targeted messaging 8	Not addressed 11
Social media	Used 4	Acknowledged, not used or studied 1	Not present 21

### 3.2 Summary of main findings: thematic results

Four themes arising from the data synthesis for this review are reported below. While these summaries draw primarily upon the 26 retained papers, additional support from the literature is identified where it was obtained as part of the overall research approach. Consistent with the intent of this review, three of the four themes address disaster response or recovery, while only the first one has a planning and preparedness focus.

#### 3.2.1 Theme one: cooperative and collaborative planning

Pre-disaster, there is a need for cooperative planning with families [e.g., (5, 37)], as well as professionals and other stakeholders (e.g., schools, utility companies etc.). Ideally communicative approaches will include children themselves as well as parents or caregivers (12) -- Sever, Sever and Vanholder say ‘listen to the children themselves’ (38). Surveys and interviews are typical consultative methods which can be employed, but Ronoh, Gaillard and Marlowe go further to give additional innovative, creative and concrete methods of involving children (39); see also sections in Mort et al. (31). Ronoh, Gaillard and Marlowe argue that the prospect of children being separated from responsible adults during times of emergency provides a good reason why they should be directly involved in planning (39). Darlington et al. indicate a prime role for parents as co-producers of their COVID-19 survey, and follow-up actions resulting from it (40).

The literature notes a lack of reliable online disaster planning resources targeting the CSHCN or CMC community. For instance, Koeffler et al. found that only 36% of resources had a focus on children with special needs; in particular there was a lack of short and concise materials, and those in languages other than English (41). Chin et al. also make a similar statement to this effect (5). These claims are consistent with So et al.’s empirical findings (42). Darlington et al. (40) and Hauesler et al. report COVID-19 survey-based data supportive of the same conclusion (43). In an Australian study, 82% of respondents felt that there was not enough COVID-19-related information targeted to children and youth with disabilities and their families (44). There is also a lack of information and communication material aimed at children themselves (42); in Australia, parents “noted a lack of resources to help explain coronavirus to children and young people with disability, such as social stories and video” (44), p. 1193.

A key point in planning is the two-way accessibility of information. This means, to begin, having patient information regularly updated and accessible to professionals and responders. For instance, the value in having portable medical info, such as the emergency information form (EIF), in both electronic and hard-copy formats recurs in several papers (13, 45–47). Privacy and data security considerations, particularly with digital information, must be respected. On the other side, parents, caregivers and children need to know how to reach their care team, including when usual channels of physical and telecommunication access are disrupted; this indicates the importance of having direct contact information, see for instance Raulgi et al. (48). There can be substantial difficulties in communicating during disaster with children having certain types of sensory or intellectual challenge (49–51).

#### 3.2.2 Theme two: pro-active outreach, engagement and response

Proactive outreach by professionals when a disaster is anticipated or occurring is recommended (13, 52). One example of a proactive approach is described by Hoffman et al., including a patient telephone contact algorithm (14); proactivity is also at least implied in the Taddei & Bulgheroni’s piece on Italy’s response to COVID-19 (53). Darlington et al. noted from survey data that many parents did feel that inadequate information was offered by their hospitals or clinical teams (40). Most post-disaster empirical papers seem to describe responses which begin with reactive communication. For instance, Dozières-Puyravel and Auvin describe parent-initiated emails preceding a COVID-19 induced transition to virtual care processes (54). Health system response also is triggered by patients showing up at hospitals (55). Gillen and Morris suggest that this is a strategy many parents may in fact have in mind as part of their own disaster response plan (11). Sakashita, Matthews, and Yamamoto argue that this is “an inadequate plan” (56). One strategy that is suggested is having a designated point person or care coordinator who is aware of service structure during a disaster and can connect parents and children to their needed care (32, 57). A Canadian study, in a non-disaster context, looked at the employment of nurse-practitioners to promote care integration for CMCs (58). In the United States, some authors suggest that CMCs should have a primary care patient medical home (10, 13) which can serve this purpose, so long as the practice is prepared for disaster response.

Information can go out by mass or individualized channels, with greater proactivity clearly required for the latter. Social media platforms straddle those boundaries perhaps. While social media has vastly expanded its role and influence in life, there has been yet limited research on its use by CMCs in disaster situations to date. So et al. note their exclusion of social media and peer forums as sources of disaster planning information as one limitation to their research (42). Rotondi et al. is one specific example of Facebook use (30). Social media is identified by parents as a channel of preferred communication (12) and has been a main source of information for parents of CMC during the COVID-19 pandemic (40). However, in the words of one parent, “sometimes having all this information on the internet is a blessing and curse” (52). Social media is also potentially a significant source of mis-information (59), as seen in the spread of ‘fake news’ related to the COVID-19 pandemic (60). The research by Darlington et al. noted that although many parents reported social media as a major source of information during the pandemic, far fewer stated that they used that information to make decisions or placed their full faith in it (40). This is consistent with the larger literature, for which a review concludes that social media is not the primary information source for most members of the public (59). However, mixed messaging from health sector sources can itself also be a problem in communicating with the caregivers of CMCs during a crisis (40, 52).

#### 3.2.3 Theme three: mobilizing and working through social networks in response

Proactive reaching out, by peers, can form the most immediate response, as for instance described in at least one Japanese case (61). Quinn & Stuart also identify the importance of personal networks as first responders (51). A similar claim is made, albeit not specific to children, by Kailes and Lollar (8). The importance of engaging neighbors is also stated by Sakashita, Matthews, and Yamamoto (56), and Rau (62). In fact, “operators and practitioners tend to rely on the relatives of people with disabilities to disseminate specific information” (30). Hassinger & Lail recommends “including functional community members” e.g., teachers, friends, etc., as part of planning (52). However, in Chin et al., focus group participants reported “difficulty in building meaningful relationships with their neighbors…. parents were unsure of their willingness to help, and did not feel empowered to start those discussions” (5), p. 192.

#### 3.2.4 Theme four: recovery

Continuity of care is important to reestablish (63) during or post-disaster, which may involve transitioning to telehealth, mHealth [mobile health], or other internet-enabled virtual communication channels, as was the case in many places where in-person care was restricted due to COVID-19 (52, 53). However, we cannot forget that not all CMCs will have ready access to the technology needed, especially during disaster disruptions; there is data on this provided by Hassinger & Lail (52) and Murphy et al. (64), as well as case discussions from European responses to COVID-19 (53, 57). Disasters also present mental health impacts, as well as disruptions to physical care and treatment. The COVID-19 pandemic has demonstrated these in the short- and medium-term (32, 53, 57). In addition, the response and recovery phases are where longer-term mental health issues, among CMCs and also their caregivers and siblings, will emerge (65, 66). These have not been extensively studied among CSHCN (47). Care teams may need to expand to adequately and fully address such issues (19, 32, 47).

Of note, re-establishing normal daily life for CMC includes resumption of disrupted schooling as well as healthcare specific programs and services. As Boon et al. state and as the COVID-19 situation has demonstrated, school closures can be “an important non-pharmaceutical component of controlling outbreaks of infectious diseases such as pandemic influenza, although little research appears to have been done on the effect of such closures” (7). This clearly matters to the children themselves: “Rather presciently [in re COVID-19], children… [with disability] in Greece drew our attention to how disruption of normal life, the impossibility of leaving the house to play or attend school, would be for them a disaster” (31), p. 157. Some additional support for this point is offered by Ducy and Stough (67). Canadian experience appears to be consistent with this as well; a survey of Canadian pediatricians reports that many CMC receive care and therapy in the school setting, and only few respondents reported that services transferred from school to home and/or community during periods of virtual learning leading to a deleterious impact on CMC (68). While multiple school years have been affected by COVID-19, parents and CMC have been able to remain in their homes through the pandemic; additional challenges are encountered where disaster destroys community infrastructure and leads to longer-term evacuation and displacement, as for instance with wildfire or flooding (20). Notably absent in the literature is any consideration of the economic well-being of families during this period of re-connection and how such social determinants of health might be addressed by the health sector and health care providers.

### 3.3 Review limitations

Disasters occur world-wide. Since this review was limited to English-language publications, its findings may be weighted toward circumstances which prevail in more highly-resourced health systems and the strategies appropriate to those contexts. As the literature we reviewed was that found at the intersection of work on children with healthcare needs, disasters and communication, we may not be aware of any insights which might be developed within studies that touch on only one of these areas or which are published in other disciplines and their specialized journals. The fact that there are few articles meeting our inclusion criteria provide a limited body of evidence, true; we cannot claim to have identified best practice *per se*, but offer several promising experience-based practices which can be refined through further research and efforts in the field.

### 3.4 Conclusions from the review

Based on the themes arising from the literature here, we offer the following conclusions, which point toward actions needed to advance current approaches to disaster communication for CMC and their families:

- Engage directly with parents/caregivers and children to advocate to policy makers the importance of establishing processes for two-way communication to prepare for disasters, with emphasis on equity despite location and language differences.
- Explore the best means for families and health care teams to leverage personal/social networks in communication.
- Implement proactive outreach, in advance of an expected disaster where lead time is available, and also in the immediate response phase. This seems easiest to do where an existing registry or inventory of the population of CMC can be deployed.
- Maintain two-way communication channels following disaster, including the use of multiple methods and redundant channels (e.g., deploy both electronic and hard-copy formats).
- Investigate and experiment with social media channels as a messaging approach; this includes efforts by reputable and trusted health care sources to counter mis-information which may be prevalent in some social media platforms. Do this in real time if possible.
- Provide information about how continuity of care will be ensured during disaster response. Virtual health services are one means by which this can be done. The COVID-19 pandemic produced a rapid outpouring of literature on this. While it seems to have largely satisfied families’ needs, there are access and equity issues. The lack of children’s presence in telehealth consult sessions, as explicitly identified in 2 studies, is worrisome insofar as we have identified the critical importance of directly engaging children/youth.
- Attend to mental health (and rehabilitation) aspects in the longer-term recovery phase; this may imply expanding the scope of the patient care team.

## 4 Discussion

The topic of communication with CMC during disaster crosses quite a heterogenous literature, which makes it challenging to synthesize. It is unclear, for instance, the extent to which varying definitions of the target population will affect the findings. It does seem safe to say that, consistent with previous reviews, the literature remains focused on preparedness, primarily employing an all-hazards approach. There is also a lack of literature and on-line resources specific to disaster preparedness and response for children with special health care needs and their families.

Overall, there is little explicit data about effective approaches to communication; this required us to ‘read between the lines’ and identify latent content related to how communication is (and is not) being addressed, the assumptions being made, and the gaps or lacuna. There are few grounds for proposing rigid set of specific best practices (do X for group Y in situation Z). Instead, illustrative vignettes can depict how disaster response might play out in particular situations. This approach was used in articles reviewed in this project (47, 49).^6^ We offer here, tailored to the context of the Canadian province of British Columbia, one future scenario of how communication with CMC might proceed during times of disaster, emergency or crisis.

*British Columbia. Late-June 2025*. A dry winter has been followed by a spring heat wave. While children are looking forward to the final weeks of school, in several small- and medium-sized communities, the fire danger has been raised to ‘extreme’, with thunderstorms and lightning in the weather forecasts. It is anticipated that uncontrolled fires may necessitate emergency evacuations.

*Planning*. Recognizing this, primary care providers (family physicians and nurse practitioners) and pediatricians whose patients include CMC put into effect the outreach plans which they have developed together with specialty care team members in case of emergency. A designated team coordinator contacts every family of CMC on the practice roster to make sure they are aware of the potential disaster, and advise (and guide) them on municipal evacuation plans. They check with the families to make sure each has its own individual disaster plan up-to-date as well, and are prepared to self-manage for a time if they may have to. The coordinators also contact mental health providers with whom they have arrangements, to confirm that their services are in place and ready to activate if needed.

*Response*. Several days of lightning and high wind combined with minimal rainfall have sparked fires across large sections of the province. Some have been successfully knocked back with aggressive actions, others are contained, but a couple of fires in steep terrain have taken off and evacuation orders have been issued for a number of communities. Time is of the essence. Clinical teams are in frantic conversation as they reach out to re-connect with families, to let them know about the status of community services. The remainder of school terms have been canceled, community health facilities are shuttered, and several family physicians are preparing to evacuate themselves.

Case coordinators keep families up-to-date with these developments, work with them to determine evacuation routes, and identify shelters which can provide key resources, such as emergency generators, medical supplies, clean water, milk for babies, and wheelchairs. Where needed, they call on contacts who understand the province-wide picture, and know which stockpiles of supplies can be moved from one site to the next. Trusted local professionals on-the-ground provide real-time updates through their official social media platforms; these complement media updates provide by health and local government sources. Families of CMC are linking with neighbors who can provide accessible transportation, satellite phone connections, and other resources.

*Recovery*. Some fires are quickly knocked down, while others rage into mid-August, putting families out of their homes for 6 weeks or more. Some communities are heavily impacted with extensive damage, others less so, but finally evacuation alerts are lifted and residents can return home. For the lucky ones, the biggest task is disposing of a freezer-full of spoiled food. In other communities, homes, schools and public facilities are gone, electric grids destroyed and running water limited or unavailable altogether. Before going anywhere, families of CMC discuss circumstances with their health provider team: where will they reside, how will they communicate with CMC, who in the vicinity can help them, which public services will resume locally and when, and which ones may be available in neighboring towns? Tele-health options have been established by many health professionals; special attention is paid to ensuring that parents and caregivers are aware of and have the resources to access these services.

Autumn comes, and things begin to return somewhat ‘back to normal’ for most – fire season is over, they have returned to their homes, and schools and other services resume. A few families, however, will remain displaced for months yet. They work with their provider teams to link to interim supports, and use the internet and other means to stay connected with the community and maintain social relationships.

Health professionals and the families discuss their experiences (with appropriate mental health supports available), and gather feedback about lessons learned and how to improve disaster response in the future.

Rather than being completely novel, our findings reinforce some important fundamental principles. Responding to disaster situations demands that all involve adhere to proactive models child and family-centered healthcare already used in the ongoing relationships between parents and caregivers, children, and health professionals. If these relationships are cultivated and running smoothly, then it should be easier for all to manage the disruptions which result from if or when disaster strikes.

## Author contributions

EL generated the initial idea and obtained research funding. NS and MD conducted the data extraction and initial analysis for the scoping review. All authors contributed to the article and approved the submitted version.

## Footnotes

^1^See for instance: https://www.mass.gov/info-details/emergency-preparedness-for-individuals-with-disabilities-and-access-and-functional

^2^https://www.ifrc.org/en/what-we-do/disaster-management/about-disasters/what-is-a-disaster/

^3^https://drexel.edu/dornsife/research/centers-programs-projects/center-for-public-health-readiness-communication/disaster-preparedness-toolkit/

^4^https://app.traumaawareschools.org/resources_public

^5^See, for instance, https://www2.gov.bc.ca/gov/content/safety/emergency-preparedness-response-recovery/preparedbc/know-your-hazards

^6^For other examples, see https://www.cdc.gov/childrenindisasters/real-stories/specialneeds/index.html
