# Supplementary material for: Communication in disasters to support families with children with medical complexity and special healthcare needs: a rapid scoping review
Source: Front Public Health. 2024 Mar 13;12:1229738. doi: 10.3389/fpubh.2024.1229738 (PMC10967951; doi:10.3389/fpubh.2024.1229738)
Supplement: Supplementary file 1 [file Data_Sheet_1.docx]

**Supplemental File: Appendix A: Search Strategies**

**Spring 2021**

## MEDLINE Search – 2021 Disaster M5

**Search was executed on:** March 21, 2021

Search Strategy:

1 disasters/ or emergencies/ or mass casualty incidents/ (62003)

2 natural disasters/ or avalanches/ or cyclonic storms/ or droughts/ or earthquakes/ or floods/ or landslides/ or tidal waves/ or tornadoes/ or wildfires/ (18963)

3 Tsunamis/ (922)

4 Volcanic Eruptions/ (1098)

5 bushfire*.mp. (339)

6 Explosions/ (4049)

7 (avalanche* or cyclone* or drought* or earthquake* or flood* or landslide* or tidal wave* or tornado* or wildfire* or bushfire* or tsunami* or hurricane* or volcan* or explosion*).tw,kw,kf. (82476)

8 biohazard release/ or chemical hazard release/ (637)

9 radioactive hazard release/ or chernobyl nuclear accident/ or fukushima nuclear accident/ (7409)

10 terrorism/ or bioterrorism/ or chemical terrorism/ or september 11 terrorist attacks/ (11021)

11 (terrorism* or bioterrorism* or terrorist*).tw,kw,kf. (9687)

12 CBRN.mp. [Chemical, biological, radiological and nuclear defense] (204)

13 ((chemical or biological or radiological or nuclear) adj3 (warfare or incident* or accident*)).tw,kw,kf. (7982)

14 disease outbreaks/ or epidemics/ or pandemics/ (144217)

15 disease transmission, infectious/ (10457)

16 emergencies/ (41209)

17 (epidemic* or pandemic*).tw,kw,kf. (185277)

18 evacuat*.mp. (22160)

19 ((national or global or community) adj5 (emerg* or outbreak*)).mp. (17117)

20 (exp hemorrhagic fevers, viral/ or hemorrhagic fever, ebola/) and outbreak*.mp. (7574)

21 ((Health* or infecti* or disease*) adj5 outbreak*).mp. (99403)

22 ((natural or health) adj5 catastrophe*).mp. (306)

23 Coronavirus Infections/ (44640)

24 Severe Acute Respiratory Syndrome/ (5490)

25 (coronavirus* or SARS* or COVID*).mp. (137295)

26 (predisaster* or disaster* or postdisaster*).tw,kw,kf. (27906)

27 (pre-disaster* or post-disaster*).tw,kw,kf. (1215)

28 or/1-27 [Disaster] (543841)

29 disabled persons/ (42959)

30 disab*.tw,kw,kf. (239768)

31 amputees/ (3667)

32 mentally disabled persons/ or mentally ill persons/ (9708)

33 Visually Impaired Persons/ (2507)

34 chronic disease/ or multiple chronic conditions/ (267324)

35 rare diseases/ (11825)

36 convalescence/ or critical illness/ (34931)

37 Catastrophic Illness/ (1055)

38 (chronic disease* or chronic health or multiple morbid* or chronic comorbid*).tw,kw,kf. (81736)

39 *Vulnerable Populations/ (5141)

40 (Life adj3 (limit* or threaten*)).mp. (101162)

41 (complex* adj3 health).tw,kw,kf. (6367)

42 (complex* adj4 (medical* or needs or problem* or condition* or patient*)).tw,kw,kf. (68223)

43 (medical* adj3 fragil*).mp. (317)

44 Palliative Care/ (55783)

45 long term illness*.mp. (774)

46 Neoplasms/ (436231)

47 Cystic Fibrosis/ (36115)

48 Cerebral Palsy/ (21145)

49 Neuromuscular Diseases/ (10369)

50 heart diseases/ (70719)

51 Dependent Ambulation/ (195)

52 parenteral nutrition, home/ (1124)

53 Parenteral Nutrition, Home Total/ (194)

54 Hemodialysis, Home/ (1986)

55 Home Infusion Therapy/ (694)

56 Ventilators, Mechanical/ (9040)

57 Tracheostomy/ (7812)

58 (Home* adj5 (ventilation or ventilator* or infusion* or hemodialysis or dialysis or parenteral)).tw,kw,kf. (6740)

59 (feed* adj3 tube*).mp. (8707)

60 (home adj3 medical device*).mp. (61)

61 ((Ventilator* or Technolog*) adj3 (assist* or depend*)).tw,kw,kf. (19687)

62 (special adj3 needs).mp. (7718)

63 exp Mental Disorders/nu [Nursing] (22157)

64 or/29-63 [Disabled persons] (1469488)

65 28 and 64 (23688)

66 infant/ or infant, newborn/ or infant, low birth weight/ or infant, postmature/ or infant, premature/ or infant, extremely premature/ (1159825)

67 child/ or child, preschool/ or children/ (1951987)

68 adolescent/ (2075740)

69 (Infan* or newborn* or new-born* or neonat* or baby* or babies or toddler* or minors* or boy or boys or boyhood or girl* or kid or kids or child* or preschool* or schoolchild* or school child or preadolescen* or adolescen* or juvenil* or youth* or teen* or under*age* or pubescen* or prepuberty* or puberty* or prepubescen* or puber*).tw,kw,kf. (2460164)

70 Pediatrics/ (55105)

71 Pediatric Nursing/ (13579)

72 (pediatr* or paediatr*).tw,kw,kf. (391183)

73 or/66-72 [Children] (4453853)

74 and/28,64,73 (5380)

75 Disabled Children/ (6471)

76 pe?diatric* cancer*.mp. (4150)

77 childhood cancer*.mp. (9064)

78 CSHCN.mp. (447)

79 or/75-78 (19036)

80 and/28,79 (226)

81 Information Dissemination/mt [Methods] (5260)

82 Needs Assessment/og [Organization & Administration] (3233)

83 Relief Work/og [Organization & Administration] (1384)

84 Health Services Accessibility/og [Organization & Administration] (7256)

85 "Delivery of Health Care"/og [Organization & Administration] (21839)

86 Risk Assessment/ (278251)

87 Disaster Planning/og [Organization & Administration] (4673)

88 postdisaster*.mp. (514)

89 (disaster* adj4 (respons* or recover* or prepar*)).tw,kw,kf. (5636)

90 "Health Services Needs and Demand"/mt, og [Methods, Organization & Administration] (2131)

91 Disaster Planning/mt, og [Methods, Organization & Administration] (6441)

92 Home Care Services/og [Organization & Administration] (7060)

93 Child Health Services/og [Organization & Administration] (5454)

94 Hospitals, Pediatric/og [Organization & Administration] (1627)

95 Emergency Medical Services/og [Organization & Administration] (8340)

96 *Health Resources/sd [Supply & Distribution] (1291)

97 Patient-Centered Care/og [Organization & Administration] (5506)

98 Social Media/ (9576)

99 social media*.tw,kw,kf. (15256)

100 facebook.mp. (4221)

101 virtual.mp. (67270)

102 twitter.mp. (3872)

103 Telemedicine/mt, og [Methods, Organization & Administration] (11929)

104 Telerehabilitation/mt, og [Methods, Organization & Administration] (300)

105 digital medicine.mp. (275)

106 digital health.mp. (2810)

107 virtual rehabilitation.mp. (150)

108 telehealth.mp. (6952)

109 (service* adj3 redesign*).mp. (464)

110 video.mp. (146601)

111 Remote Consultation/ (5090)

112 ((remote* or Virtual*) adj4 care).tw,kw,kf. (2864)

113 (care adj4 coordinat*).mp. (12463)

114 or/81-113 [Communication & Support] (610027)

115 og.fs. [Organization & Administration] (492658)

116 28 and 64 and 73 and (114 or 115) (714)

117 28 and 79 and (114 or 115) (54)

118 116 or 117 (726)

119 limit 118 to yr="1995 -Current" (696)

120 limit 119 to english language (677)

121 remove duplicates from 120 (675)

122 comment/ or letter/ or news/ (1747518)

123 121 not 122 (658)

124 limit 123 to (systematic reviews pre 2019 or systematic reviews) (34)

125 limit 123 to "review articles" (120)

126 or/124-125 [Reviews] (141)

127 123 not 126 (517)

128 Caregivers/ (38902)

129 Family/ (77740)

130 parents/ or fathers/ or mothers/ or single parent/ (116305)

131 or/128-130 (219536)

132 28 and 114 and 73 and 131 (244)

133 limit 132 to yr="1995 -Current" (236)

134 limit 133 to english language (232)

135 comment/ or letter/ or news/ (1747518)

136 134 not 135 (225)

137 136 not 123 (181)

138 limit 137 to (systematic reviews pre 2019 or systematic reviews) (3)

139 limit 137 to "review articles" (17)

140 138 or 139 (19)

141 137 not 140 [Remaining] (162)

## CINAHL Search – Disaster CIN1

**Date:** March 23, 2021

| **#** | **Query** | **Results** |
| --- | --- | --- |
|  | Limit to Academic Journals | 389 |
| S113 | S110 AND S111 Limiters - Exclude MEDLINE records | 431 |
|  | Limit to Academic Journals | 985 |
| S112 | S110 AND S111 | 1,049 |
| S111 | LA English | 7,571,853 |
| S110 | S108 AND S109 | 1,058 |
| S109 | PY 1995-2021 | 7,227,776 |
| S108 | **S103** OR **S107** | 1,076 |
| **S107** | S24 AND S52 AND S60 AND S100 AND S106 | 70 |
| S106 | S104 OR S105 | 126,033 |
| S105 | (MH "Mothers+") OR (MH "Fathers") OR (MH "Parents") | 91,826 |
| S104 | (MH "Caregivers") | 36,890 |
| **S103** | S101 OR S102 | 1,076 |
| S102 | S24 AND S68 AND 100 | 1 |
| S101 | S24 AND S52 AND S60 AND S100 | 1,076 |
| S100 | S70 OR S71 OR S72 OR S73 OR S74 OR S75 OR S76 OR S77 OR S78 OR S79 OR S80 OR S81 OR S82 OR S83 OR S84 OR S85 OR S86 OR S87 OR S88 OR S89 OR S90 OR S91 OR S92 OR S93 OR S94 OR S95 OR S96 OR S97 OR S98 OR S99 | 516,046 |
| S99 | TI (care N4 coordinat*) OR AB (care N4 coordinat*) | 9,898 |
| S98 | ( TI ((remote* or Virtual*) N4 care) ) OR ( AB ((remote* or Virtual*) N4 care) ) | 1,954 |
| S97 | TI video OR AB video | 34,379 |
| S96 | TI (service* N3 redesign*) OR AB (service* N3 redesign*) | 468 |
| S95 | TI telehealth OR AB telehealth | 4,735 |
| S94 | TI virtual rehabilitation OR AB virtual rehabilitation | 469 |
| S93 | TI digital health OR AB digital health | 2,299 |
| S92 | TI digital medicine OR AB digital medicine | 622 |
| S91 | ( TI (social media* or twitter or facebook) ) OR ( (social media* or twitter or facebook) ) | 28,848 |
| S90 | (MH "Remote Consultation") OR (MH "Telerehabilitation") OR (MH "Telenursing") OR (MH "Telepsychiatry") OR (MH "Teleradiology") OR (MH "Telepathology") OR (MH "Telemedicine") OR (MH "Telehealth") | 27,251 |
| S89 | (MH "Social Media") OR (MH "Facebook") OR (MH "Twitter") | 16,901 |
| S88 | (MH "Patient Centered Care") OR (MH "Continuity of Patient Care") OR (MH "Patient Care") OR (MH "Patient Education/MT/OG") OR (MH "Health Education/MT") OR (MH "Preventive Health Care/MT/PC") | 95,628 |
| S87 | (MH "Health Resource Allocation/MT") OR (MH "Health Resource Utilization/MT") | 371 |
| S86 | (MH "Emergency Medical Service Communication Systems") OR (MH "Emergency Service Information Systems") OR (MH "Education, Emergency Medical Services") OR (MH "Emergency Medical Services+") | 109,187 |
| S85 | (MH "Hospitals, Pediatric) | 9,925 |
| S84 | (MH "Health Services for Persons with Disabilities/MT/OG") OR (MH "Community Health Services") OR (MH "Community Health Nursing+") OR (MH "Community Mental Health Services+") OR (MH "Community Networks") OR (MH "Family Services") OR (MH "Rehabilitation, Community-Based") OR (MH "Maternal Health Services+") OR (MH "Health Services, Indigenous") | 105,603 |
| S83 | (MH "Child Health Services/MT/OG") | 100 |
| S82 | (MH "Home Health Care+/OG/MT") | 1,154 |
| S81 | (MH "Disaster Planning+/MT/PC") | 1,066 |
| S80 | (MH "Health Services Needs and Demand+/MT") | 5 |
| S79 | ( TI (disaster* N4 (respons* or recover* or prepar*)) ) OR ( AB (disaster* N4 (respons* or recover* or prepar*)) ) | 3,703 |
| S78 | TI postdisaster* OR AB postdisaster* | 261 |
| S77 | (MH "Medical Countermeasures") OR (MH "Disaster Planning+/OG/MT") OR (MH "Mass Casualty Training") | 1,487 |
| S76 | (MH "Risk Assessment") | 113,420 |
| S75 | (MH "Health Services Accessibility/MT") OR (MH "Health Care Delivery/MT") | 5,548 |
| S74 | (MH "Humanitarian Aid+/OG") | 48 |
| S73 | (MH "Needs Assessment/OG") | 2,414 |
| S72 | (MH "Needs Assessment/OG") | 2,414 |
| S71 | (MH "Health Information Networks/OG/MT") OR (MH "Home Health Care Information Systems/OG") | 12 |
| S70 | (MH "Selective Dissemination of Information/OG/MT") | 3 |
| S69 | S24 AND S68 | 283 |
| S68 | S62 OR S63 OR S64 OR S65 OR S66 OR S67 | 26,985 |
| S67 | TI CSHCN OR AB CSHCN | 323 |
| S66 | TI childhood cancer* OR AB childhood cancer* | 4,690 |
| S65 | ( TI (pediatric* cancer* OR peadiatric cancer*) ) OR ( AB (pediatric* cancer* OR peadiatric cancer*) ) | 2,655 |
| S64 | (MH "Childhood Neoplasms") | 4,967 |
| S63 | (MH "Parents of Disabled Children") | 4,552 |
| S62 | (MH "Child, Medically Fragile") OR (MH "Child, Disabled") | 13,506 |
| S61 | S24 AND S52 AND S60 | 5,101 |
| S60 | S54 OR S55 OR S56 OR S57 OR S58 OR S59 | 1,297,957 |
| S59 | ( TI (pediatr* or paediatr*) ) OR ( AB (pediatr* or paediatr*) ) | 147,946 |
| S58 | (MH "Maternal-Child Nursing") OR (MH "Pediatric Nursing+") | 24,625 |
| S57 | (MH "Pediatrics+") | 21,856 |
| S56 | TI ( (Infan* or newborn* or new-born* or neonat* or baby* or babies or toddler* or minors* or boy or boys or boyhood or girl* or kid or kids or child* or preschool* or schoolchild* or school child or preadolescen* or adolescen* or juvenil* or youth* or teen* or under*age* or pubescen* or prepuberty* or puberty* or prepubescen* or puber*) ) OR AB ( (Infan* or newborn* or new-born* or neonat* or baby* or babies or toddler* or minors* or boy or boys or boyhood or girl* or kid or kids or child* or preschool* or schoolchild* or school child or preadolescen* or adolescen* or juvenil* or youth* or teen* or under*age* or pubescen* or prepuberty* or puberty* or prepubescen* or puber*) ) | 815,090 |
| S55 | (MH "Adolescence") OR (MH "Adolescent, Hospitalized") | 547,297 |
| S54 | (MH "Infant, Premature") OR (MH "Infant, Postmature") OR (MH "Infant, Low Birth Weight+") OR (MH "Infant, High Risk") OR (MH "Infant, Drug-Exposed") OR (MH "Infant+") OR (MH "Child+") OR (MH "Infant, Hospitalized") OR (MH "Infant, Newborn+") | 692,258 |
| S53 | S24 AND S52 | 21,697 |
| S52 | S25 OR S26 OR S27 OR S28 OR S29 OR S30 OR S31 OR S32 OR S33 OR S34 OR S35 OR S36 OR S37 OR S38 OR S39 OR S40 OR S41 OR S42 OR S43 OR S44 OR S45 OR S46 OR S47 OR S48 OR S49 OR S50 OR S51 | 1,319,910 |
| S51 | (MH "Mental Disorders+") | 590,814 |
| S50 | TI (special N3 needs) OR AB (special N3 needs) | 7,814 |
| S49 | ( TI ((Ventilator* or Technolog*) N3 (assist* or depend*)) ) OR ( AB ((Ventilator* or Technolog*) N3 (assist* or depend*)) ) | 8,969 |
| S48 | TI (home N3 medical device*) OR AB (home N3 medical device*) | 48 |
| S47 | TI (feed* N3 tube*) OR AB (feed* N3 tube*) | 3,972 |
| S46 | ( TI (Home* N5 (ventilation or ventilator* or infusion* or hemodialysis or dialysis or parenteral)) ) OR ( AB (Home* N5 (ventilation or ventilator* or infusion* or hemodialysis or dialysis or parenteral)) ) | 3,224 |
| S45 | (MH "Tracheostomy") | 4,586 |
| S44 | (MH "Ventilators, Mechanical") | 3,101 |
| S43 | (MH "Home Intravenous Therapy") | 1,541 |
| S42 | (MH "Heart Diseases+") | 291,316 |
| S41 | (MH "Neuromuscular Diseases") | 2,370 |
| S40 | (MH "Cerebral Palsy") | 12,596 |
| S39 | (MH "Cystic Fibrosis") | 8,154 |
| S38 | (MH "Neoplasms") | 84,895 |
| S37 | TI long term illness* OR AB long term illness* | 1,301 |
| S36 | (MH "Palliative Care") | 37,556 |
| S35 | TI medical* N3 fragil* OR AB medical* N3 fragil* | 322 |
| S34 | TI ( (complex* N4 (medical* or needs or problem* or condition* or patient*)) ) OR AB ( (complex* N4 (medical* or needs or problem* or condition* or patient*)) ) | 26,210 |
| S33 | TI complex* N3 health OR AB complex* N3 health | 5,490 |
| S32 | TI ( (Life N3 (limit* or threaten*)) ) OR AB ( (Life N3 (limit* or threaten*)) ) | 26,724 |
| S31 | (MM "Special Populations") | 3,032 |
| S30 | TI ( chronic disease* or chronic health or multiple morbid* or chronic comorbid* ) OR AB ( chronic disease* or chronic health or multiple morbid* or chronic comorbid* ) | 94,231 |
| S29 | (MH "Recovery") | 34,344 |
| S28 | (MH "Rare Diseases") | 1,631 |
| S27 | (MH "Critical Illness") OR (MH "Catastrophic Illness") OR (MH "Chronic Disease+") | 80,786 |
| S26 | TI disab* OR AB disab* | 126,291 |
| S25 | (MH "Disabled") OR (MH "Amputees") OR (MH "Mentally Disabled Persons") | 44,601 |
| S24 | S1 OR S2 OR S3 OR S4 OR S5 OR S6 OR S7 OR S8 OR S9 OR S10 OR S11 OR S12 OR S13 OR S14 OR S15 OR S16 OR S17 OR S18 OR S19 OR S20 OR S21 OR S22 OR S23 | 161,400 |
| S23 | TI ( predisaster* or disaster* or postdisaster* ) OR AB ( predisaster* or disaster* or postdisaster* ) | 14,009 |
| S22 | TI ( coronavirus* or SARS* or COVID* ) OR AB ( coronavirus* or SARS* or COVID* ) | 42,528 |
| S21 | (MH "Coronavirus Infections") OR (MH "COVID-19") OR (MH "Middle East Respiratory Syndrome") OR (MH "Severe Acute Respiratory Syndrome") | 25,823 |
| S20 | TI ( ((natural or health) N5 catastrophe*) ) OR ( ((natural or health) N5 catastrophe*) ) | 122 |
| S19 | TI ( ((Health* or infecti* or disease*) N5 outbreak*) ) OR AB ( ((Health* or infecti* or disease*) N5 outbreak*) ) | 6,898 |
| S18 | MH "Hemorrhagic Fevers, Viral+" AND outbreak* | 2,601 |
| S17 | TI ( (national or global or community) N5 (emerg* or outbreak*) ) OR AB ( (national or global or community) N5 (emerg* or outbreak*) ) | 7,990 |
| S16 | TI evacuat* OR AB evacuat* | 4,521 |
| S15 | (MH "Emergencies") | 10,631 |
| S14 | TI ( epidemic* or pandemic*) OR AB ( epidemic* or pandemic*) | 49,991 |
| S13 | TI infectious N5 disease transmission OR AB infectious N5 disease transmission | 398 |
| S12 | (MH "Disease Transmission, Horizontal") | 955 |
| S11 | (MH "Disease Outbreaks+") | 40,949 |
| S10 | TI ( (chemical or biological or radiological or nuclear) N3 (warfare or incident* or accident*) ) OR AB ( (chemical or biological or radiological or nuclear) N3 (warfare or incident* or accident*) ) | 1,090 |
| S9 | TI CBRN OR AB CBRN | 73 |
| S8 | TI ( terrorism* or bioterrorism* or terrorist* ) OR AB ( terrorism* or bioterrorism* or terrorist* ) | 4,239 |
| S7 | (MH "Terrorism") | 6,476 |
| S6 | TI nuclear N5 accident* OR AB nuclear N5 accident* | 319 |
| S5 | (MH "Chemical Hazard Release") OR (MH "Biohazard Release") | 319 |
| S4 | TI ( avalanche* or cyclone* or drought* or earthquake* or flood* or landslide* or tidal wave* or tornado* or wildfire* or bushfire* or tsunami* or hurricane* or volcan* or explosion* or bushfire* ) OR AB ( avalanche* or cyclone* or drought* or earthquake* or flood* or landslide* or tidal wave* or tornado* or wildfire* or bushfire* or tsunami* or hurricane* or volcan* or explosion* or bushfire* ) | 13,128 |
| S3 | (MH "Fires+") OR (MH "Wildfires") | 5,480 |
| S2 | (MH "Natural Disasters") | 12,067 |
| S1 | (MH "Disasters") OR (MH "Emergency Evacuation") OR (MH "Mass Casualty Incidents") | 9,778 |

**Summer 2020**

**Search strategies:**

**Ovid Medline**

1. adolescent/ or child/ or child, preschool/ or infant/ or infant, newborn/

2. (infant or baby or babies or toddler* or preschool* or child or "child's" or children* or childhood or boy or boys or boyhood or girl or girls or girlhood or adolescen* or preadolescen* or kid or kids or prepuberty* or puberty* or prepubescen* or puber* or pubescen* or teen* or youth*).ti,ab.

3. Pediatrics/

4. Pediatric Nursing/

5. (pediatr* or paediatr*).ti,ab.

6. parents/ or fathers/ or mothers/ or single parent/

7. (parent* or parents).ti,ab.

8. Legal Guardians/

9. Minors/

10. (guardian* or parent or parents).ti,ab.

11. 1 or 2 or 3 or 4 or 5 or 6 or 7 or 8 or 9 or 10

12. disabled persons/ or chronic disease/ or multiple chronic conditions/

13. (disab* or chronic disease* or chronic health or multiple morbidities).ti,ab.

14. (complex needs or complex health needs or complex health care needs or complex healthcare needs or complex medical needs or complex health conditions or complex medical conditions).ti,ab.

15. (medical complexity or medical complexities or medically complex or medical fragility or medically fragile).ti,ab.

16. chronic comorbid*.ti,ab.

17. persons with hearing impairments/ or hearing disorders/ or blindness/ or deaf-blind disorders/ or deafness/

18. (visual impair* or hearing impair* or deaf* or blind* or hard of hearing).ti,ab.

19. home care services/ or home health nursing/ or home nursing/ or home care services, hospital-based/ or home health aides/

20. ((home health or Home care) adj3 (agenc* or service* or provider* or nurse* or nursing or patient*)).ti,ab.

21. parenteral nutrition/ or parenteral nutrition, total/ or hemodialysis, home/ or home care services, hospital-based/ or home infusion therapy/ or parenteral nutrition, home/ or parenteral nutrition, home total/ or ventilators, mechanical/ or tracheostomy/

22. (Home* adj5 (ventilation or ventilator* or infusion* or hemodialysis or dialysis or parenteral or tube feed*)).ti,ab.

23. pediatricians/

24. (pediatrician* or paediatrician*).ti,ab.

25. (technolog* adj3 (assist* or depend*)).ti,ab.

26. (home medical device* or special healthcare or special health care or special needs).ti,ab.

27. 12 or 13 or 14 or 15 or 16 or 17 or 18 or 19 or 20 or 21 or 22 or 23 or 24 or 25 or 26

28. disabled children/

29. (cshcn or cshcns or cmc or cmcs or yshcn or yshcns).ti,ab.

30. 28 or 29

31. 11 and 27

32. 30 or 31

33. communication/ or health communication/ or information dissemination/

34. (communicat* or correspond* or information or contact or contacting or contacted or contacts).ti,ab.

35. patient education as topic/ or social media/

36. (texting or text messag* or SMS or app or apps or mobile application or Internet* or online).ti,ab.

37. ((disrupt* or recover* or reestablish* or resum* or restor* or access* or receipt) adj5 (service* or care or network* or system or systems)).ti,ab.

38. needs assessment/

39. (assess* adj2 needs).ti,ab.

40. 33 or 34 or 35 or 36 or 37 or 38 or 39

41. disasters/ or emergencies/ or mass casualty incidents/ or natural disasters/ or avalanches/ or cyclonic storms/ or droughts/ or earthquakes/ or floods/ or landslides/ or tidal waves/ or tornadoes/ or wildfires/

42. terrorism/ or bioterrorism/ or chemical terrorism/ or mass casualty incidents/ or september 11 terrorist attacks/

43. Fukushima nuclear accident/

44. epidemics/ or pandemics/

45. (predisaster or postdisaster or disaster or disasters).ti,ab.

46. CBRN.ti,ab.

47. (bioterror* or terror* or evacuat* or epidemic or epidemics or pandemic or pandemics).ti,ab.

48. 41 or 42 or 43 or 44 or 45 or 46 or 47

49. disaster planning/

50. 48 or 49

51. 32 and 40 and 50

52. (coronavirus or coronaviruses or 2019-nCov or SARS-CoV-2 or nCOV or COVID).ti,ab.

53. 30 and 52

54. 53 or 51

**CINAHL**

S1 (MH "Adolescence+") OR (MH "Child+") OR (MH "Infant") OR (MH "Infant, Hospitalized") OR (MH "Infant, Newborn+")

S2 TI ( infant or baby or babies or toddler* or preschool* or child or "child's" or children* or childhood or boy or boys or boyhood or girl or girls or girlhood or adolescen* or preadolescen* or kid or kids or prepuberty* or puberty* or prepubescen* or puber* or pubescen* or teen* or youth ) OR AB ( infant or baby or babies or toddler* or preschool* or child or "child's" or children* or childhood or boy or boys or boyhood or girl or girls or girlhood or adolescen* or preadolescen* or kid or kids or prepuberty* or puberty* or prepubescen* or puber* or pubescen* or teen* or youth )

S3 (MH "Pediatrics")

S4 (MH "Pediatric Nursing")

S5 TI ( pediatr* or paediatr* ) OR AB ( pediatr* or paediatr* )

S6 (MH "Parents") OR (MH "Fathers") OR (MH "Mothers") OR (MH "Single Parent") OR (MH "Parents of Disabled Children")

S7 TI ( parent or parents* ) OR AB ( parent or parents* )

S8 (MH "Guardianship, Legal")

S9 (MH "Minors (Legal)")

S10 TI ( guardian* or parent or parents ) OR AB ( guardian* or parent or parents )

S11 S1 OR S2 OR S3 OR S4 OR S5 OR S6 OR S7 OR S8 OR S9 OR S10

S12 (MH "Chronic Disease+")

S13 TI ( disab* or chronic disease* or chronic health or multiple morbidities ) OR AB ( disab* or chronic disease* or chronic health or multiple morbidities )

S14 TI ( complex needs or complex health needs or complex health care needs or complex healthcare needs or complex medical needs or complex health conditions or complex medical conditions ) OR AB ( complex needs or complex health needs or complex health care needs or complex healthcare needs or complex medical needs or complex health conditions or complex medical conditions )

S15 TI ( medical complexity or medical complexities or medically complex or medical fragility or medically fragile ) OR AB ( medical complexity or medical complexities or medically complex or medical fragility or medically fragile )

S16 TI chronic comorbid* OR AB chronic comorbid*

S17 (MH "Hearing Disorders+") OR (MH "Blindness+")

S18 TI ( visual impair* or hearing impair* or deaf* or blind* or hard of hearing ) OR AB ( visual impair* or hearing impair* or deaf* or blind* or hard of hearing )

S19 (MH "Home Health Care+")

S20 TI ( (home health or Home care) N3 (agenc* or service* or provider* or nurse* or nursing or patient*) ) OR AB ( (home health or Home care) N3 (agenc* or service* or provider* or nurse* or nursing or patient*) )

S21 (MH "Parenteral Nutrition+") OR (MH "Ventilators, Mechanical") OR (MH "Respiratory Therapy+") OR (MH "Tracheostomy Care")

S22 TI ( Home* N5 (ventilation or ventilator* or infusion* or hemodialysis or dialysis or parenteral or tube feed*) ) OR AB ( Home* N5 (ventilation or ventilator* or infusion* or hemodialysis or dialysis or parenteral or tube feed*) )

S23 (MH "Pediatricians")

S24 TI ( pediatrician* or paediatrician* ) OR AB ( pediatrician* or paediatrician* )

S25 TI ( technolog* N3 (assist* or depend*) ) OR AB ( technolog* N3 (assist* or depend*) )

S26 TI ( home medical device* or special healthcare or special health care or special needs ) OR AB ( home medical device* or special healthcare or special health care or special needs )

S27 S12 OR S13 OR S14 OR S15 OR S16 OR S17 OR S18 OR S19 OR S20 OR S21 OR S22 OR S23 OR S24 OR S25 OR S26

S28 (MH "Child, Disabled")

S29 TI ( cshcn or cshcns or cmc or cmcs or yshcn or yshcns ) OR AB ( cshcn or cshcns or cmc or cmcs or yshcn or yshcns )

S30 S28 OR S29

S31 S11 AND S27

S32 S30 OR S31

S33 (MH "Communication")

S34 TI ( communicat* or correspond* or information or contact or contacting or contacted or contacts ) OR AB ( communicat* or correspond* or information or contact or contacting or contacted or contacts )

S35 (MH "Patient Education") OR (MH "Social Media+")

S36 TI ( texting or text messag* or SMS or app or apps or mobile application or Internet* or online ) OR AB ( texting or text messag* or SMS or app or apps or mobile application or Internet* or online )

S37 TI ( (disrupt* or recover* or reestablish* or resum* or restor* or access* or receipt) N5 (service* or care or network* or system or systems) ) OR AB ( (disrupt* or recover* or reestablish* or resum* or restor* or access* or receipt) N5 (service* or care or network* or system or systems) )

S38 (MH "Needs Assessment")

S39 TI assess* N2 needs OR AB assess* N2 needs

S40 S33 OR S34 OR S35 OR S36 OR S37 OR S38 OR S39

S41 (MH "Disasters") OR (MH "Emergency Evacuation") OR (MH "Fires") OR (MH "Wildfires") OR (MH "Mass Casualty Incidents") OR (MH "Natural Disasters")

S42 (MH "Terrorism") OR (MH "Bioterrorism")

S43 TI ( september 11 OR fukushima nuclear OR avalanche* OR hurricane* OR drought OR droughts OR eathquake OR landslide OR tidal wave* OR tornado* OR wildfire* OR natural disaster* )

S44 TI ( epidemic OR epidemics OR pandemic* ) OR AB ( epidemic OR epidemics OR pandemic* )

S45 TI ( predisaster or postdisaster or disaster or disasters ) OR AB ( predisaster or postdisaster or disaster or disasters )

S46 TI CBRN OR AB CBRN

S 47 TI ( bioterror* OR terror* OR evacuat* ) OR AB ( bioterror* OR terror* OR evacuat* )

S48 S41 OR S42 OR S43 OR S44 OR S45 OR S46 OR S47

S49 MH "Disaster Planning"

S50 S48 OR S49

S51 S32 AND S40 AND S50

S52 TX coronavirus OR coronaviruses OR 2019-nCov OR SARS-CoV-2 OR nCOV OR COVID

S53 S30 AND S52

S54 S51 OR S53

Total results: 284

**Grey literature**

- Google Scholar (“Medical complexity” OR “medically complex” OR “medically fragile” OR “medical fragility” OR “special health care needs") AND (disaster OR epidemic OR pandemic)
  - NOTE: COVID not included as search term, as many article pages advertised COVID results, and this generated many irrelevant results
  - First 100 results reviewed
- MedRxiv (“Medical complexity”; “medically complex”; “medically fragile”; “medical fragility”; “special health care needs”; CMC; CYSHCN; YSHCN)
  - No relevant results
- TRIP
  - First 100 results reviewed; no additional results found
- PEDro (“Medical complexity”; “medically complex”; “medically fragile”; “medical fragility”; “special health care needs”; CMC; CYSHCN; YSHCN)
- OAIster (“Medical complexity”; “medically complex”; “medically fragile”; “medical fragility”; “special health care needs”; CMC; CYSHCN; YSHCN)
- Google: (“Medical complexity” OR “medically complex” OR “medically fragile” OR “medical fragility” OR “special health care needs") AND (disaster OR epidemic OR pandemic)
  - First 100 results reviewed

**Supplemental File: APPENDIX** **B**

**Summaries of relevant articles, with emphasis on what they say about communication**

| **Authors & Date** | **Title** | **Summary** |
| --- | --- | --- |
| Asher & Pollak, 2009 | *Planning Emergency Evacuations for Students with Unique Needs-- Role of Occupational Therapy* | This study focused on disaster and emergency planning at schools, using case examples. The tool is a written individualized evacuation plan, the nature of which is described in some detail (which may help inform development of other communication materials). As one part of the document, specific key and backup contacts are identified. Family members and professionals are directly involved in plan development. Notes that CMCs may have specific challenges in communicating, which need to be accounted for. This article addresses only a moment in time during an actual disaster, and ends at the point where students have been evacuated from a school building. |
| Baker et al, 2012 | *Preparing families of children with special health care needs for disasters: an education intervention* | This study was undertaken with a convenience sample of parents/guardians of children with chronic medical conditions seen at one US children’s hospital. The intervention was a one-to-one education session, which consisted of discussion plus distribution (in paper copy) of informational handouts. Content was generic rather than tailored to individual medical conditions. Authors favour this approach over mass media, which they suggest has unknown effectiveness (though not cited); this approach mobilizes “informal social context”. Healthcare professionals presumed to be trusted sources of information. |
| Cacioppo et al, 2021 | *Emerging health challenges for children with physical disabilities and their parents during the COVID-19 pandemic: The ECHO French survey* | This nation-wide survey describes an array of channels used to promote the survey (email and social media; via parent groups, advocacy groups and professional networks). Major impacts include loss of social contacts leading to negative moods and behavioural problems, and disruption in education and health care services. Parents reported greatest concerns with rehabilitation rather than medical issues (perhaps reflective of the degree of disability, but not stated). Parents have greatly increased burden, having to perform a lot of services usually done by skilled professionals. Discusses need/value of creating a ‘care coordinator’ position for clients. |
| Chin et al, 2020 | *A mixed-method analysis: Disaster preparedness of families with children with access and functional needs* | A questionnaire and focus group study with 20 parents of CAFN (children with access and functional needs) in California. Consistent with other literature, found relatively low levels of preparedness, though parents anticipated several types of disasters were more likely than not to occur. Communication as an issue was raised with respect to schools, as well as neighbours. Does not address any recommendations or suggestions for improvement however. |
| Darlington et al, 2021 | *COVID-19 and children with cancer: Parents’ experiences, anxieties and support needs* | Reports a UK-based survey of parents of children with cancer (N=171), initiated by 2 health centres and circulated through networks of professional organizations, health charities. (Notes that these groups regularly updated and disseminated the latest Covid advice through their channels.) Parents were involved in developing the instrument. Two parts: close-ended and open-ended. Emphasis on social media; for instance, notes that parents were expressing fears via social media groups. In the quantitative data, it was observed that 49% got information from their clinical team, while 84% got information from social media; smaller numbers of people stated that social media was influencing their decisions (25%) and few trusted it (8%). In the qualitative content analysis (N=130), it is noted that some parents avoided news or social media as a coping strategy. Other qualitative results were that parents found a lack of information targeted to children with disabilities (rather than the general adult population), and that they found their teams/hospitals not to be providing enough information; more guidance and support was desired. Mixed or changing messaging was also a problem. Among the sub-group who responded with open feedback, the main sources of information were health charities, clinical staff, and the news. Concludes by stating that the feedback was directly translated into action, for instance through co-creation of additional information distributed through the same professional organization, charity and local provider networks (not described in detail). |
| Dozieres-Puyravel et al, 2021 | *Usefulness, limitations, and parental opinion about teleconsultation for rare pediatric epilepsies* | This reports on use of telehealth in one French hospital centre during the COVID-19 pandemic. Notes that the service was completely not set up prior to the pandemic to do any virtual health consultations. Contacts appear to involve parents initially reaching out by email, with questions or issues for the care team; by the time virtual services were set up, most had done this. Most done by audiovisual, some by phone largely due to difficulties with the technology, or parents’ fear/anxiety. Suggests looking for ways to substitute lack of physical exams and observation. Noted that the children often were not part of the sessions, reasons unexplained. |
| Dursun et al, 2020 | *Caring for the Most Vulnerable: A Model for Managing Maladaptive Behavior in Children with Mental Special Needs During the COVID-19 Pandemic* | Focus on children with mental impairments (e.g., autism) in Turkey. Built an all-new nation-wide system involving care coordinators with videoconferencing, and local “psychosocial intervention teams” [new type of provider] for more complex cases. Based on a mobile app with 24/7 live response. Unclear why the parents regular care team was not a first point of contact? The service was advertised with a news conference and information to local provider organizations; presumably media but no additional details. Social isolation etc. due to Covid giving parents concern with maladaptive behaviours; a new concern and one more pressing than physical health. |
| Goodhue et al, 2016 | *Mixed-Methods Pilot Study: Disaster Preparedness of Families with Children Followed in an Intestinal Rehabilitation Clinic* | This study focused on disaster planning, with earthquakes as a particular example. Research involved a convenience sample with a survey and 2 focus groups; English only. As far as preparation, a majority had some form of back-up for power and medical supplies, but almost none had an emergency information form (EIF). Communication was anticipated by parents to be them reaching out, by attending a hospital when supplies were exhausted. However, they also reported from past experience that professionals unfamiliar with their children’s conditions could be unhelpful. Having direct contact information was important (physicians, also pharmacy, insurance, utility cost.) It was seen as helpful if professionals gave written letters or other information detailing medical conditions/needs. Communication also incidentally in the research through peer-to-peer interaction in the focus groups, with some parents sharing preparedness ideas. |
| Haeusler et al, 2021 | *Managing low-risk febrile neutropenia in children in the time of COVID-19: What matters to parents and clinicians* | This study is in context of efforts to develop a home-based program in Australia for children with febrile neutropenia [cancer]. The relevant portion is results of a parent survey (n=14) using an adaptation of the ECOM (Effective Communication in Outbreak Management for Europe) instrument. Findings: “Communication that parents wanted to receive about COVID-19 included information about ‘chance COVID-19 is serious for child’ (n = 7), how is it treated (n = 7), safe return to school (n = 5) and illness prevention (n = 5). Communication around mode of transmission, incubation and symptoms were infrequently identified as important factors. All parents reported they preferred information provided by their oncologist, followed by state/federal governments. Qualitative comments highlighted a need to address the availability of information tailored to children with cancer including those off treatment, as well as more consideration given to return to school advice for regions with higher community transmission”. |
| Hassinger and Lail, 2021 | *PANDEMIC IS DECLARED: Early Experience from Families of Children with Medical Complexity during SARS-COV-2 Lockdown: Information to Drive System Change* | This is largely a story of one family’s experience, with some additional literature and recommendations for systems change. Themes include supply chain disruptions; communication challenges, including mixed messaging; behavioural health impacts; disruptions to school-based and other allied health services; and use of telehealth as a substitute for in-person care. Recommends proactive outreach in a couple of spots, and the importance of multimodal communication. |
| Hoffman et al, 2018 | *Hurricane Irma Impact on the Inpatient Population at a Tertiary Children's Hospital in Florida* | This is a case study of how one Children’s tertiary care centre, in Orlando FL, responded to a hurricane event. The hospital remained open; it sheltered 13 patients in place and received 13 patient transfers. This covers planning and response. Notes that patients needed both scheduled and unscheduled care for conditions not related to the hurricane itself. Proactively identified vulnerable patients (those dependent on medical devices) and set aside space and staff for them. Notes that parents otherwise planned desperate measures, such as faking illness at a community hospital (p1397). |
| Kaziny, 2014 | *The Prehospital Care of Children with Special Health Care Needs* | One section of this paper deals with disasters, covering both preparedness and response; the preparedness section largely summarizes other papers we have identified. There is a call for co-creation of EIFs. Also, “The prehospital provider should take a proactive role in reaching out to families with CSHCN in the case of a disaster to provide detailed information regarding the community's plan for disaster shelters and evacuation assistance.” Pre-hospital provider is never clearly defined, but mostly seems to be EMT and transport. |
| Kobayashi et al (2016) | *The lack of antiepileptic drugs and worsening of seizures among physically handicapped patients with epilepsy during the Great East Japan Earthquake* | A survey of patients affected with epilepsy in the aftermath of a 2011 earthquake. Patients attend a Children’s Rehabilitation Centre, though not all seem to be children. It is stated that hospital also provides transitionary care for younger adults; the median age of those surveyed was 14, through the range was 4-38 (n=161, out of 279 eligible). Noted that telecommunication systems were down for 5 days; during this post-earthquake period, 29% of respondents ran out of needed medications, and of those, 46% were unable to contact the hospital centre. Important information about alternative means of accessing prescription medicine was conveyed by website; effectiveness of this was not assessed. |
| Mace et al, 2010 | *Pediatric issues in disaster management, part 3: special healthcare needs patients and mental health issues.* | Summarizes some literature on CSHCNs and disasters. Like others, notes importance of EIF [and hard copy in the event of power failure], of engaging with community stakeholders (e.g., utility companies) for disaster planning. Conversations can be phrased using a requirements/gap analysis strategy. Suggests ‘backpack’ tag with info for children who may become separated from caregivers (e.g., during school evacuations). Use of ‘message maps’. Mapping of patient home location using Geographic Information Systems. One of a series of 4 articles (but maybe the only one with this focus). |
| Mort et al, 2020 [Edited book] | *Children and young people’s participation in disaster risk reduction: Agency and Resilience* | This edited collection reports the CUIDAR project (Cultures of Disaster Resilience Among Children and Young People), a pan-European project implemented in 5 countries, which conducted Dialogues with Children using a rights-based framework. The Greek component (n=63) specifically involved children with sensory (vision, hearing) and multiple disabilities. 3D modeling is a technique described (p124). |
| Murray, 2011 | *Disaster preparedness for children with special healthcare needs and disabilities* | Mostly addresses preparedness; refers to a systematic review though this paper is presented as ‘Ask the expert’ rather than a traditional academic article reporting those results. Argues for parental involvement with professionals in developing emergency plans, and that children should have direct access to materials at an appropriate developmental level, sharing feelings and asking questions. Notes there may be misinformation (in context of those with cognitive impairment particularly). Recommends use of an emergency information form, per the AAP 2010 policy statement; ideally in electronic form as paper records can be lost, and where parents can’t access usual professionals, emergency departments may not have access to medical records during disaster. Addresses long-term psychosocial aspects. Potential separation from parents during disaster. Challenge of coordinating multiple specialists. Role of utility companies. |
| Nakayama et al, 2014 | *Effect of a blackout in pediatric patients with home medical devices during the 2011 eastern Japan earthquake* | This study focused on post-disaster treatment. Communication explicitly identified as problematic in earthquake aftermath for these families. Research involved review of medical records, and patient questionnaire. Communication involved patients reaching out by showing up at regional medical centre (which escaped damage) due to being without power/supplies or running out of them. Often this was unannounced as patients unable to make contact. Transport also identified as a problem due to gasoline shortages (84%). Recommends re. communication for preparedness (1) info sharing among medical centres and local government; (2) portable personal medical information documentation; (3) availability of contact numbers. Text and cellphone communication reportedly superior to regular telephone in the post-earthquake conditions (though this is literature from another study and not investigated here). |
| Quinn, 2010 | *Disaster Preparedness* | This article is directed at speech-language pathologists and focuses upon children with “complex communication needs” (which may or may not overlap with CMC, unclear). The paper provides summary findings from a literature review, and a sparsely detailed description of a ‘community outreach’ program related to children with CCN. The audience appears to be parents and/or first responders (‘community stakeholders’), but unclear. Literature review notes importance of personal social networks. |
| Raulji et al, 2018 | *Impact of Hurricane Katrina on healthcare delivery for New Orleans patients, 2005–2014* | A children’s hospital in New Orleans surveyed patients in the pediatric hematology and cancer program one-year post Hurricane Katrina; on the basis of feedback a Hurricane Action Plan was developed and implemented, and a follow-up survey conducted in 2012-13 with patients seen during those years, to assess program impacts. In 2006, not knowing where to get care, if the hospital was open [it was], and how to contact specialist professionals, were barriers to accessing care. The Action Plan involved providing patients with a treatment-during-disaster/evacuation roadmap and contact information. Evaluation found that, at the time of the second survey, "Only 29 (36%) had their roadmap/treatment plan available with them … [and] Although emergency contact for the child's hematology/oncology staff had been given to all the patients, only 57 (72%) reported they had the information available with them." Communication seems to involve providing pamphlets. Providing info to families on flash drives was recommended by some survey respondents. |
| Ronoh et al, 2015; 2017 | *Children with disabilities and disaster preparedness: A case study of Christchurch*  AND  *Bridging the Participatory Gap: Children with Disabilities and Disaster Risk Reduction* | Two papers: the first is a case study on working with children with disabilities in one Christchurch school on disaster response planning activities; the second extends the work to two additional schools. Provides concrete and detailed description of tools that can be used to engage children with disabilities in disaster planning. The 2015 paper uses Christchurch earthquakes of 2010 and 2011 as specific background, with comments on telecommunications issues that arose. |
| Rotondi, 2019 | *Facebook page created soon after the Amatrice Earthquake for deaf adults and children, families, and caregivers provides an easy communication tool and social satisfaction in maxi-emergencies* | Notes that there was limited data to identify the relevant population that was or might be affected (the lack of a unified standard definition of CMC suggests a parallel issue). Authors created a page in the aftermath of the disaster; prioritized timely translation of official and credible information. Concludes that, “a well-received social question-answer service could help to spread information on safety practices in a day-to-day, easy, and affordable way”. Includes children as target audience but not specifically focused on them. |
| Shimada and Funato, 1995 | *Home mechanical ventilation in the aftermath of the Hanjin-Awaji earthquake disaster* | A questionnaire study of caregivers 1-month post-earthquake. Peer-to-peer communication features prominently. “During the first days immediately afterwards, neighbors were the most helpful persons available. After the earthquake, the importance of the establishment and maintenance of good communication with neighbors became more important than ever”. Also important was role of the Baku Baku club (network among parents of CMCs), which appears to proactively reach out to members and to bring resupplies. Recommends a registration system for home care patients. |
| So et al, 2020 | *An Evaluation of the Literacy Demands of Online Natural Disaster Preparedness Materials for Families* | This paper attempted to identify free, online disaster preparedness learning materials which Americans would be likely to access, and to assess the literacy demands of these. A sub-section of the analysis is devoted to materials specifically directed to parents of CSHCN. This constituted 5% of the total (18 out of 356 websites). While the authors found overall the materials to be adequate to poor in terms of five measures of literacy level, materials specific to CSHCN fared somewhat better. Authors note that no materials are directed to children themselves as a target audience, and their research did not include direct parent/child perspectives, or capture other potential sources of online information such as social media. Interestingly, healthcare organizations did not produce or maintain any of the internet sources. |
| Stallwood et al, 2006 | *Assessing Emergency Preparedness of Families Caring for Young Children with Diabetes and Other Chronic Illnesses* | This article advocates the importance of children with chronic diseases to use medical alert jewelry or other wearables, which can provide key information to responders during times of emergency. A number of methods of promoting this are mentioned. For instance, for children themselves, “posters, coloring books, age-appropriate video presentations in the waiting room, and the like can be displayed, promoting the important messages of emergency preparedness, along with examples of how to meet the needs of preparing such devices”. Further, “Information sources specific for the caregiving adult should also be readily available. These sources may include brochures outlining Internet resources, a list of local vendors carrying the necessary equipment, informational videos related to the assembly and maintenance of emergency medical supplies, and the display of various medical alert identification schemes and a sample emergency kit.” These are largely one-way communication; conversations (assisted by checklists) are also recommended for increasing individual preparedness. |
| Taddei and Bulgheroni, 2020 | *Facing the real time challenges of the COVID-19 emergency for child neuropsychology service in Milan* | Most of the children here have developmental disabilities. Used phone contacts until telehealth services were ready, appears to be proactive, “we reach out to our patients” about the changes in hospital access being made. Did not require patients to download any app. Some technology issues with accessibility, and language barriers. Also notes the frequent absence of children from the sessions, reasons not explained. The pandemic is creating new issues for parents, more psychosocial and child behavior ones than the normal physical health matters dealt with during the former in-person visits. |
